# Bellevue Hospital Notes

**Published:** 1873-11

**Authors:** 


BELLEVUE HOSPITAL NOTES.

*Night Sweats.*—In connection with phthisis, empyema, etc., have been more successfully treated with $\frac{1}{256}$ grain doses of sulphate of atropia at bed-time than by any other-sized dose administered.

The size of the dose was accidental, but the results realized have been most gratifying. It is usually administered in cinnamon water.

*Peritonitis.*—The importance of not relying upon pain as a symptom of peritonitis was again illustrated in a patient in whom the disease was not suspected, until post-mortem revealed its presence.

Absence of pain in this disease is of not infrequent occurrence.

*Pneumonia.*—Several cases of pneumonia were in the wards at this writing, and all are doing very well. Quinine, Carbonate of Ammonia for its stimulant effect, and whisky or brandy, were the remedies chiefly relied upon in the cases seen. Quinine, when given for its apyogenic effect, it is said, must be given in large doses, and the patients receive, accordingly, 25 or 30 grains per day, and when a pneumonia is treated in this way a majority of the patients get well. When given in ordinary doses of five grains three times a day, a majority of the patients get well, and that without the administration of the remedy with any special reference to its apyogenic influence. "Who is who, and which is what?" might be a query with an outside observer.

*Laxatives.*—A new remedy has been introduced as a laxative, which is said to be preferable to many of the salines on account of its agreeable taste. It is the Sulphovinate of Soda in two-drachm doses.

Another very efficient and much-used laxative compound is the following:

℞. Ext. Colocynth co....................grs. vi.

Ol. Caryophyl......................gtts. ij.

M.

Div. in pilulæ No. ij.

*Chronic Bronchitis and Emphysema.*—An old man, who is the subject of this combination of disease, derives great comfort when suffering from the intense paroxysmal dyspnœa which commonly accompanies it, by taking drachm doses of

℞. Hoffman's anodyne,
M. Sol. Morphia (U. S.) aa at the commencement of a paroxysm, and repeating it p. r. n.

